# Feasibility and accuracy of a robotic guidance system for navigated spine surgery in a hybrid operating room: a cadaver study

**DOI:** 10.1038/s41598-020-64462-x

**Published:** 2020-05-05

**Authors:** Gustav Burström, Marcin Balicki, Alexandru Patriciu, Sean Kyne, Aleksandra Popovic, Ronald Holthuizen, Robert Homan, Halldor Skulason, Oscar Persson, Erik Edström, Adrian Elmi-Terander

**Affiliations:** 1 Department of Clinical Neuroscience, Karolinska Institutet, Stockholm, Sweden; 2 Department of Neurosurgery, Karolinska University Hospital, Stockholm, Sweden; 3 Philips Research North America, Cambridge, USA; 4 Department of Image Guided Therapy Systems, Philips Healthcare, Best, the Netherlands; 5 Department of Neurosurgery, Landspitali University Hospital, Reykjavik, Iceland

**Keywords:** Neuropathic pain, Spinal cord diseases, Three-dimensional imaging, Outcomes research

## Abstract

The combination of navigation and robotics in spine surgery has the potential to accurately identify and maintain bone entry position and planned trajectory. The goal of this study was to examine the feasibility, accuracy and efficacy of a new robot-guided system for semi-automated, minimally invasive, pedicle screw placement. A custom robotic arm was integrated into a hybrid operating room (OR) equipped with an augmented reality surgical navigation system (ARSN). The robot was mounted on the OR-table and used to assist in placing Jamshidi needles in 113 pedicles in four cadavers. The ARSN system was used for planning screw paths and directing the robot. The robot arm autonomously aligned with the planned screw trajectory, and the surgeon inserted the Jamshidi needle into the pedicle. Accuracy measurements were performed on verification cone beam computed tomographies with the planned paths superimposed. To provide a clinical grading according to the Gertzbein scale, pedicle screw diameters were simulated on the placed Jamshidi needles. A technical accuracy at bone entry point of 0.48 ± 0.44 mm and 0.68 ± 0.58 mm was achieved in the axial and sagittal views, respectively. The corresponding angular errors were 0.94 ± 0.83° and 0.87 ± 0.82°. The accuracy was statistically superior (p < 0.001) to ARSN without robotic assistance. Simulated pedicle screw grading resulted in a clinical accuracy of 100%. This study demonstrates that the use of a semi-automated surgical robot for pedicle screw placement provides an accuracy well above what is clinically acceptable.

## Introduction

The insertion of pedicle screws remains one of the critical steps in spine surgeries involving thoracolumbar posterior fixation. The procedure poses risks for complications, as both neural and vascular structures are in close proximity to the pedicles. A meta-analysis by Gelalis *et al*. reported that 1-6.5% of pedicle screws placed using free-hand technique had a cortical violation >4 mm^1^. Meanwhile, minimally invasive spine (MIS) surgery is increasingly preferred due to reductions in blood loss, length of hospital stay, and surgical site infections^2^. Accuracy and complication rates, however, are not different^3,4^. When relying only on fluoroscopy in MIS, the pedicle perforation rate has been reported to be in the range of 12.5–13.5%^5–7^. Since MIS reduces anatomical feedback to the surgeon, it can be argued that image guidance, and perhaps also robotic guidance, has a natural role in this kind of surgery^8^.

The promises of increased accuracy, decreased complication rates, and reduced radiation exposure to the surgical team has led to an increasing use of robots in MIS surgery^9^. There are currently several navigated robotic systems available on the market employing various strategies for 3D planning using pre- or intraoperative fluoroscopy or CT. The most frequently reported ones in the literature are the Mazor robots (MAZOR Robotics Ltd., Caesarea, Israel) and the ROSA Spine system (Medtech S.A., Montpellier, France). The Mazor robots rely on integrating preoperative computed tomography (CT) and 3D-planning with intraoperative fluoroscopy. Intraoperative fluoroscopy or CT is used for 3D planning in the ROSA system, which also utilizes a navigation camera and reference markers attached to the patient and two attached to the robot, which allows for real-time instrument tracking. Both systems employ a robotic arm for instrument guidance^10^. Reviews on robot-guided surgery conclude that the technology consistently yields an acceptable, but not improved, level of accuracy compared to fluoroscopy-based techniques^10,11^. A more recent randomized study, however, showed superior accuracy^12^. Effects on radiation exposure, length of stay, and operative time remain unclear^10,11,13,14^.

Previous studies have reported the development of a surgical navigation system based on augmented and virtual reality navigation in a hybrid operating room (OR) setup^15–19^. A clinical accuracy of 89% without, and 100% with instrument tracking, in MIS surgery has previously been demonstrated with the augmented reality surgical navigation system (ARSN)^17,18^. The next step to improve accuracy even further, would be to eliminate the surgeon’s manual error by adding robot-assistance during the procedure. However, investing in a navigated surgical robot needs to be cost-effective, improving accuracy without prolonging surgery^20^. In this study, we examine the integration of a surgical robot with the ARSN system. Jamshidi needles were placed in the thoracic, lumbar and first sacral pedicles of four cadavers, using a minimally invasive approach. We investigate whether the addition of a robotic arm to identify and maintain entry position and planned trajectory, increases accuracy and if it adds surgical time compared to non-robot-assisted pedicle screw placement using the same ARSN system and reported rates for robot-assisted surgery in the literature. Using the ARSN, the radiation exposure to the staff is negligible and no lead aprons are required^21,22^.

## Materials and Methods

The study was conducted in compliance with ethical guidelines for human cadaver studies. This study did not involve the collection of identifiable private information and is exempt from the need for ethical approval under the United States legislation number 45 CFR § 46.102. Informed consent for donation to scientific research had been signed before death by the donors or after death by relatives, according to the local guidelines approved by the University of Cincinnati College of Medicine. Four cadavers (ages 55-94, 3 females, 1 male) without spinal pathologies, were used in this study. All procedures were performed in a hybrid OR equipped with ARSN technology (Philips Healthcare, Best, The Netherlands), as previously described^21^. All surgeries were performed by neurosurgeons experienced with spinal surgery and the ARSN system. Using a minimally invasive approach, Jamshidi needles were placed in 78 thoracic, 31 lumbar and 4 sacral pedicles from Th2 to S1. One hundred and sixteen pedicles were included in the study; 3 lumbar pedicles were not possible to attempt due to insufficient length of the Jamshidi needles for the MIS procedure.

### The robot arm

**Figure 1:**
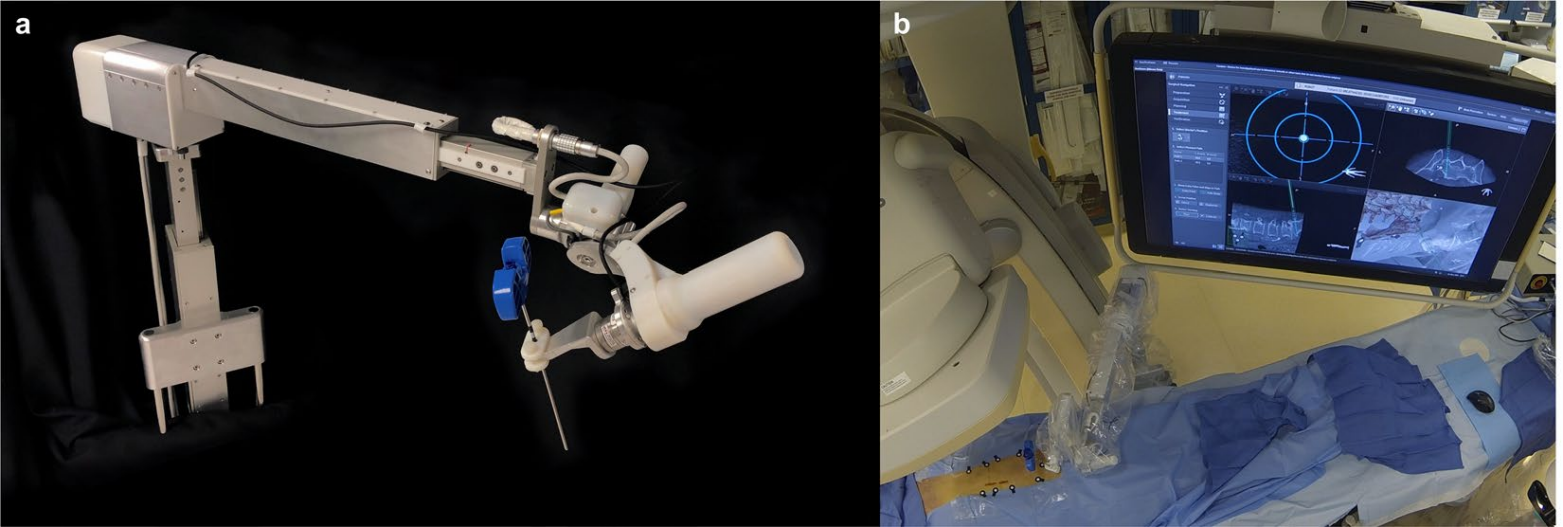
Layout of the robotic arm with a Jamshidi needle (blue) in the instrument adapter is depicted in (**a**). In (**b**), the entire surgical setup in the operating room is seen with the robot, C-arm and navigation interface on a monitor.

The robotic guidance system comprises a robotic arm integrated into the hybrid OR as seen in Fig. 1. The robot is lightweight (7 Kg), and directly mounted on the OR table. The robot features 5 active and one passive joint (J) and is comprised of two units, one with 3 degrees of freedom (DOF), and the other with 2 DOF. The 3DOF translation module includes vertical linear stage (J0), shoulder revolute joint (J1), and horizontal linear stage (J2). It carries the 2DOF rotation module that comprises two spherical linkages: J3 and J4, both of which have angles of 65 degrees, with J3 mounted at 60 degrees to vertical. The system automatically aligns the elected instrument according to the surgical plan using only instrument tracking feedback without need for the tracking of the robot itself. After identifying the levels to be treated by fluoroscopy, the robot is mounted on the table rail system in less than a minute. The robot is immediately operational without any need for calibration due to the integration to the ARSN. Its workspace covers at least 6 spinal levels and it is manually positioned by sliding the robot along the table rail. The robot arm has 5 motorized axes used for positioning of instruments in 3 translations and two angles of rotation, for positioning of straight instruments such as needles and drills. An integrated force-torque sensor is used to measure loads on the instrument guide. This enables data collection of forces exerted on the patient, as well as force control of the robot, where the user “drives” the robot by directly pushing/pulling on the instrument guide. The robot can also be controlled remotely via a gaming-type controller or move autonomously with feedback from the surgical navigation system. The instrument guide on the robot accepts adapters for standard surgical instruments, such as drills, Jamshidi needles, pedicle probes, etc.

### Robot-guided pedicle screw cannulation

The cadavers were placed in the prone position, with the head in a Mayfield clamp, on a Maquet surgical table (Alphamaquet 1150, Maquet AG, Switzerland) connected to a motorized ceiling-mounted C-arm system (AlluraClarity FD20, Philips Healthcare, Best, The Netherlands) in a hybrid OR. The ARSN system is based on video input from four optical cameras in the frame of the flat detector^16–18^. Video from all cameras is automatically co-registered with the coordinate system of the C-arm.

Between 8 and 12 sterile, flat, adhesive circular markers were placed on the skin around the surgical site. These markers form the basis of patient tracking and position recognition for the system, without the need for a dynamic reference frame^18,21^. A previous clinical study has demonstrated that the patient tracking is not affected by breathing movements^15^. A 3D Cone Beam Computed Tomography (CBCT) was performed to capture the desired spinal levels (XperCT, Philips Healthcare). Typically 4 to 6 spinal levels are imaged using the 48 cm field of view imaging mode used in this study^22^. The C-arm rotates 180° in 8 seconds during acquisition, creating a 3D volume with 0.45 mm voxel size, as has been previously described^17^. The spine was subsequently segmented with identification of the vertebral pedicles and displayed as a 3D volume in addition to multiplanar slice display^23^. Based on the automatic identification of the pedicles, screw paths were suggested by the system and the surgeon verified and adjusted them as needed before insertion^23^.

**Figure 2:**
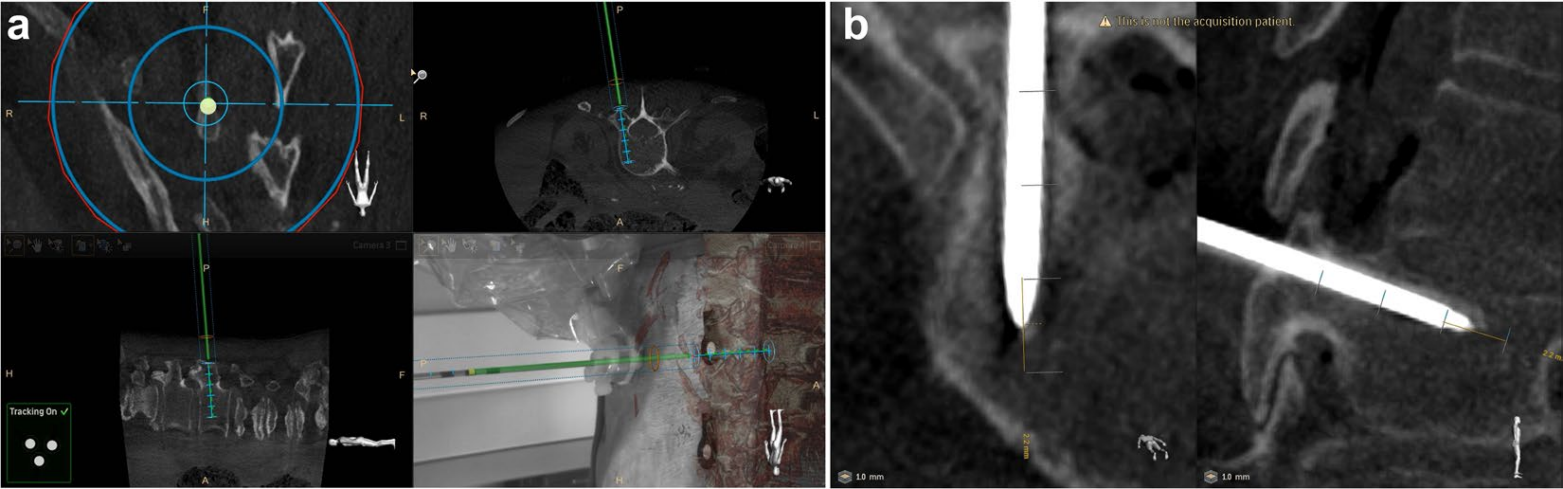
Depiction of the surgical navigation interface during robot assisted surgery: In (**a**), top left shows a coronal view along the pedicle trajectory, top right shows an axial view with the navigated Jamshidi in green, bottom left a sagittal view, and bottom right a camera feed with an augmented reality overlay depicting the underlying anatomy. In (**b**), a post-operative computed tomography scan of a placed Jamshidi needle is depicted. Axial view to the left and sagittal view to the right.

The optical and intraoperative images were automatically registered with no user interaction required. The use of the robotic arm did not affect the setup time in this regard as the steps are identical to regular use of the ARSN. The system provides two modes of navigation: augmented reality or virtual reality. For augmented reality, the C-arm was automatically rotated into one of four possible positions where one optical camera was aligned along the planned axis of insertion. The other three optical cameras provided angulated views for the alignment of the surgical instruments. The augmented reality and video feedback of the surgical site aided in fast skin entry localization and in performing skin incisions for the minimally invasive approach. For the subsequent insertion of the Jamshidi needle (152 mm in length and 3.0 mm in diameter) (CareFusion, Franklin Lakes, NJ) to the bone entry point, the virtual reality mode was utilized which used all cameras for tracking of the needle. In this feature, the system localizes a marker sticker with a high-contrast pattern of black and white lines attached to the shaft of the instrument and calculates the location of the instrument axis and tip relative to the patient and the surgical plan. In this setup, the Jamshidi needle was tracked and placed in the working channel of the robot instrument guide as seen in Fig. 2. Thus, the robot itself is not tracked and instead its accuracy relies solely on the tracking information provided by the ARSN system regarding position of the patient and surgical instrument. After the skin incision was made, the robot was instructed to align the tracked Jamshidi needle with the intended path. The Jamshidi needle, placed through the working channel of the robot’s instrument guide, was hammered into the bone entry point and further into the pedicle until the desired depth was reached, with continuous device position feedback from the navigation system. The ARSN was used to continuously monitor the device position to avoid skiving or other deviations from the planned path. Verification CBCT images were acquired at bone entry point and final Jamshidi needle position, and used for accuracy analysis. For illustration and conceptual purposes, K-wires were also placed in some pedicles. There was no X-ray fluoroscopy needed during placement of the Jamshidi needles. The insertion time, defined as the time required from skin incision to final Jamshidi needle placement, was measured. Insertion times were excluded if they were influenced by factors not related to the experiments. The time for repositioning the robot when it could not reach the next intended pedicle was also measured. For accuracy measurements, verification CBCT-scans were acquired at bone entry point and at the final Jamshidi needle position. In addition, snapshots of the navigation software display, showing progress along the intended navigational path, were taken for comparison and illustration purposes as depicted in Fig. 2.

### Technical and Clinical Accuracy Evaluation

To reduce metal artifacts in the 3D images, verification CBCT-scans were performed with 30 degrees angulation towards the inserted device to avoid collinearity with the X-ray beam. The positions of the inserted Jamshidi needles relative to the planned paths were measured on these postoperative scans to assess technical accuracy and reported as axial (i.e. mediolateral direction) or sagittal (i.e. caudo-cranial direction) deviations as highlighted in Fig. 2b, or as the compound 2D distance^16,24,25^. Likewise, the angular deviation was measured in the axial and sagittal planes^15,25–27^.

Gertzbein grading of the Jamshidi needle positions was performed by extrapolating the Jamshidi diameters to common pedicle screw diameters, as has previously been published^18,28^: grade 0 (within the pedicle without cortical breach), grade 1 (up to 2 mm breach, minor perforation including cortical encroachment), grade 2 (more than 2 and up to 4 mm breach, moderate breach), and grade 3 (more than 4 mm breach, severe displacement)^29^. Clinical accuracy was defined as a maximum cortical breach of <2 mm, corresponding to Gertzbein grades 0 and 1^29^. A screw thickness was assumed to have been chosen if the pedicle width was at least as wide as the screw diameter, otherwise the next-widest screw diameter was assumed to have been used. The grading was performed on combined axial, sagittal, and coronal views.

### Statistical Analysis

Insertion time, accuracy measurement, and angular deviations were expressed as means ± standard deviations and as medians [interquartile ranges]. A Wilcoxon rank sum test was calculated to identify whether there was a correlation between consecutive cadavers and time (i.e. a sign of learning curve). A two-tailed Welch Two Sample t-test was used for testing statistical significance between the current results and our previously published accuracy data using ARSN without robotic guidance. Statistical analysis was performed using RStudio (RStudio Team (2016). RStudio: Integrated Development for R. RStudio, Inc., Boston, MA). A *p*-value less than 0.05 was considered as significant.

## Results

**Figure 3:**
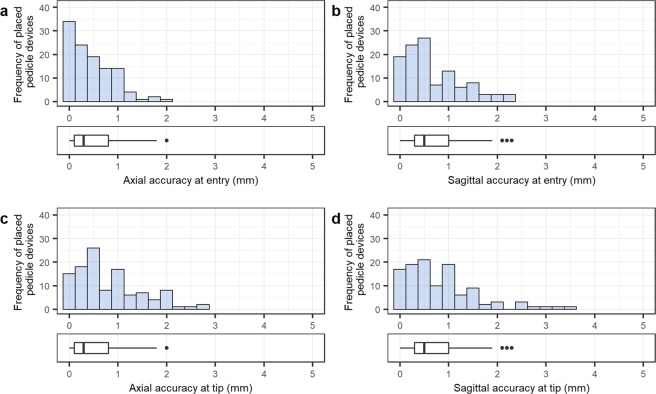
Distribution of distances between placed pedicle devices (n = 113) and planned paths showing: accuracy at bone entry point in the axial (**a**) and sagittal (**b**) view, and accuracy at device tip in the axial (**c**) and sagittal (**d**) view. Boxplots display the median as a solid line, hinges corresponding to upper and lower quartiles, and whiskers indicating data up to 1.5 interquartile range. Dots represent outliers.

**Figure 4:**
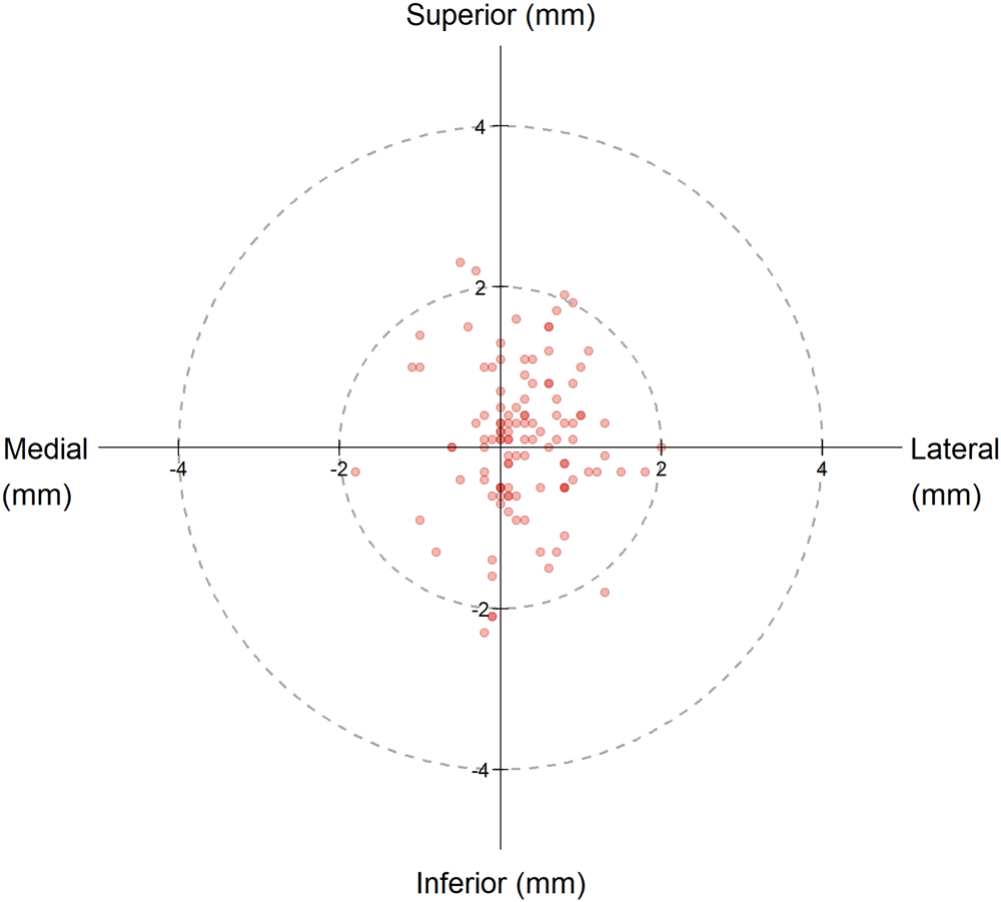
Entry point errors of the Jamshidi needles (n = 113). Planned entry point is the center of the circle and red dots represent the deviation, in mm, of each Jamshidi needle placed.

The mean distances between the 113 placed Jamshidi needles and the planned paths are illustrated in Fig. 3. At bone entry point, the technical accuracy was 0.48 ± 0.44 mm (0.30 [0.10–0.80] mm) and 0.68 ± 0.58 mm (0.50 [0.30–1.0] mm) in the axial and sagittal plane, respectively. At the tip, the mean technical accuracy was 0.82  ±  0.66 mm (0.60 [0.3–1.1] mm) in the axial plane and 0.83 ± 0.73 mm (0.60 [0.3–1.2] mm) in the sagittal plane. The mean compound (2D) technical accuracy at bone entry point was 0.94  ±  0.59 mm (0.89 [0.5–1.4] mm). No Jamshidi entry point deviated >2.4 mm from the plan, and 92% of entry points were within 2 mm from the plan (Fig. 4).

**Figure 5:**
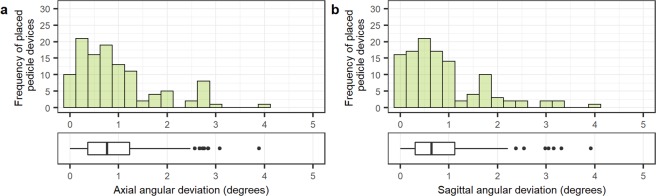
Distribution of angle errors between placed pedicle devices (n = 113) and planned paths, showing angle deviations in the axial (**a**) and sagittal (**b**) view. Boxplots display the median as a solid line, hinges corresponding to upper and lower quartiles, and whiskers indicating data up to 1.5 interquartile range. Dots represent outliers.

The mean angular deviation between the bone entry of the navigated device and the planned path was 0.94  ±  0.83° (0.76 [0.37–1.20] °) and 0.87  ±  0.82° (0.65 [0.30–1.10] °) in the axial and sagittal plane, respectively. The spread of angular deviations is shown in Fig. 5.

**Figure 6:**
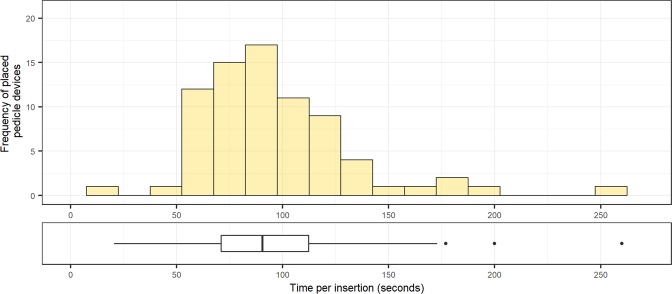
The distribution of insertion times per pedicle cannulation (n = 76), including both robotic navigation and pedicle cannulation with Jamshidi needle. Boxplots display the median as a solid line, hinges corresponding to upper and lower quartiles, and whiskers indicating data up to 1.5 interquartile range. Dots represent outliers.

The mean navigation time, from skin incision to placed Jamshidi needle following a virtual planned path, was 96 ± 37 seconds (90 [71–110] s, Fig. 6). There were no statistically significant differences between consecutive cadavers (p > 0.05 for all). The time to reposition the robot, every 5 levels, was 106 ± 71 seconds (60 [40–220] seconds).

By extrapolating Jamshidi needle thickness to simulate pedicle screws at different diameters, a simulated clinical grading according to Gertzbein was performed, as shown in Table 1. Irrespective of screw diameter used, up to the maximum of 7 mm tested, a clinical accuracy of 100% was achieved. At screw diameters up to 7 mm, 20 screws would have been grade 1 and the remaining 93 screws grade 0. The mean pedicle width excluding sacral pedicles was 7.3 ± 2.3 mm (6.9 [5.8–8.7] mm) and the proportion of thoracic pedicles was 69%.

**Table 1:** Simulated clinical grading of pedicle screws according to Gertzbein grade, at different max screw diameters. Gertzbein grade 0 and 1 are considered clinically accurate.

	Gertzbein Grade 0 (no. of screws)	Gertzbein Grade 1 (no. of screws)	Gertzbein Grade 2 (no. of screws)	Gertzbein Grade 3 (no. of screws)	Clinical Accuracy
4-mm screw	103	10	0	0	100%
6-mm screw	95	18	0	0	100%
7-mm screw	93	20	0	0	100%

The technical accuracy was compared to our own previously published data using the ARSN system and found to be statistically superior (Table 2). Comparisons were made to a human cadaver MIS study with navigated path (p < 0.001)^17^, a clinical trial on open deformity surgery with navigated path (p < 0.001)^15^, and a porcine cadaver MIS study with instrument tracking (p < 0.001)^18^.

**Table 2:** Studies reporting technical accuracy rates for spine surgery with lumbosacral and thoracic pedicle screw placement using the augmented reality surgical navigation system (ARSN).

Study	Surgical technique	Navigation type	Performed on	Devices placed	No. of pedicles	Clinical accuracy, Gertzbein 0-1 (%)	Technical accuracy, mean error in 2D (mm)	Statistical significance versus technical accuracy of current study
Elmi-Terander *et al*., 2016^16^	Open	Navigated path	Cadaver	Screws	47	40/47 (85%)	nd	nd
Elmi-Terander *et al*., 2018^17^	MIS	Navigated path	Cadaver	Jamshidi	66	nd	2.2 ± 1.3	p < 0.001
	MIS	Navigated path	Cadaver	Screws	18	16/18 (89%)	nd	nd
Elmi-Terander *et al*., 2018^15^	Open	Navigated path	Patients	Screws	253	238/253 (94.1%)	1.97 ± 1.33	p < 0.001
Burström *et al*., 2019^18^	MIS	Instrument tracking	Porcine	Jamshidi	78	76-78/78 (100, 97.4, 97.4)*	1.7 ± 1.0	p < 0.001
Current study	MIS	Instrument tracking + surgical robot	Cadaver	Jamshidi	113	113/113 (100%)*	0.94 ± 0.59	

Data are presented as absolute numbers, percentages or mean ± standard deviation. Abbreviations: MIS = Minimally invasive surgery, nd = no data. *Simulated Gertzbein grading: screw diameters extrapolated from Jamshidi needle center to represent up to 4, 6, or 7 mm screws.

## Discussion

In this study, we present the first validation of a new investigational robotic arm specifically designed for a hybrid OR and integrated with an ARSN system. We find a very high accuracy as shown in bone entry deviation data and angular errors (0.48  ±  0.44 mm, 0.94  ±  0.83° and 0.68 ± 0.58 mm, 0.87  ±  0.82°) in the axial and sagittal planes, respectively. This is in line with top performers among previously published material on bone entry deviation accuracy and superior in regard to angular errors (Table 3)^30–36^. This in turn resulted in a favorable simulated Gertzbein grading (100% accuracy), well in line with the vast majority of recent studies that typically report 98 – 100% accuracy^34,37–41^. Notably, the results of this study indicate a superior clinical, as well as technical accuracy, for the ARSN system with robotic guidance compared to previous results when the same ARSN system is used without robotic assistance (Table 2)^15–19^.

**Table 3:** Studies reporting technical accuracy rates for spine surgery with lumbosacral and thoracic pedicle screw placement using robotic guidance.

Study	Robot type	No. of pedicles	Study design	Instrumented spine	Technical accuracy, at entry (mm)	Angulation error (°)
Togawa, 2007^30^	SpineAssist (Mazor)	32	MIS cadaver setup	Lumbar	1.01 ± 0.64 (axial) 0.96 ± 0.62 (sagittal)	
Czerny, 2015^31^	iSYS1 (Interventional systems)	20	MIS cadaver setup	Thoracic, lumbar	0.35 (axial) 0.7 (sagittal)	
Fujishiro, 2015^32^	Renaissance (Mazor)	216	Open surgery cadaver setup	Thoracic, lumbosacral	0.64 ± 0.59 (axial) 0.77 ± 0.62 (sagittal)	
Lefranc, 2015^33^	ROSA (Medtech)	38	MIS cadaver setup	Thoracic, lumbosacral	2.05 ± 1.2 (2D distance)	
Van Dijk, 2015^34^	SpineAssist (Mazor)	178	Retrospective MIS patient cohort	Lumbar	2.0 ± 1.2 (2D distance)	2.2 ± 1.7 (axial) 2.9 ± 2.4 (sagittal)
Croissant, 2018^35^	MAXIO (Perfint)	24	MIS cadaver setup	Thoracic, lumbar	1.2 ± 0.9 (axial) 0.5 ± 0.9 (sagittal)	
Jiang, 2018^36^	ExcelsiusGPS (Globus)	8	Retrospective open surgery patient cohort	Lumbar	3.2 [0.9-5.4] (2D distance)	2.4 [0.7-3.8] (2D angle)
Current study	(Philips)	113	MIS cadaver setup	Thoracic and lumbosacral	0.48 ± 0.44 (axial) 0.68 ± 0.58 (sagittal)	0.94 ± 0.83 (axial) 0.87 ± 0.82 (sagittal)

Data are presented as mean ± standard deviation. Abbreviations: MIS = Minimally invasive surgery.

Recent meta-analyzes have shown reliable, but not superior, clinical accuracy of robotic guidance when directly compared to pedicle screw placement using standard fluoroscopic guidance^10,11,13^. On the other hand, a meta-analysis on postoperative revision rates by Staartjes *et al*., showed lower rates among navigation and robot-guided surgeries compared to free hand surgeries^13^. This highlights the complexity of choosing a good outcome parameter for robot-guided surgery and the possible shortcomings of clinical grading scales alone. Therefore, it is our belief that there is an added benefit in reporting all available accuracy data and not solely rely on either clinical grading scales or absolute, technical accuracies for new technologies, when outcome data such as revision rates or complication rates are not available.

Previous studies on the accuracy of surgical guidance systems found that a key issue in accurate pedicle screw placement is the correct approach and entry into the pedicle^18,42,43^. This is partly determined by a well-chosen bone entry point, devoid of ridges and slopes, which may deflect the instrument used to penetrate the bone^44^. Surgical navigation with 3D-representation of the deep bone anatomy adequately addresses the identification of a good bone entry point and the augmented reality visualization is especially helpful in this respect^18^. Another aspect of the same problem is the surgeon’s ability to maintain the correct position and angle of approach on the bone entry point^10,17^. The addition of a surgical robot arm is expected to rectify this issue. Nonetheless, vigilance in maintaining the robotic-guided screw trajectories has been cautioned by other authors. Issues mentioned include altered trajectories due to soft tissue pressures, forceful surgical application, and bone surface skiving^10^. We found no instances where the system had failed to maintain the desired trajectory to within 2.4 mm. The slight lateral deviation seen in Fig. 4 is probably due to the downward slope of the lamina directing the instrument downwards and hence laterally^44^. Based on our findings in this study we suggest that surgical robot-guidance with simultaneous instrument tracking is well suited to maintain position and angulation as the bone entry point is accessed and that it provides sufficient feedback on any unwanted deviation from planned trajectory. The use of an augmented and virtual reality interface further supports this, as any deviation during insertion would be apparent to the surgeon, thus providing the possibility to stop and correct the trajectory if needed. The integrated force-torque sensor of the current robot could also be part of a feedback system to warn the surgeon if the device deviated from the planned path due to pressure from soft tissue or bone.

Even though the robot used in this study was a prototype yet to be optimized for workflow and speed, the mean navigation time following the virtual planned path was 96 ± 37 seconds from skin incision to placement of a Jamshidi needle. In a previously published study using the same navigation system without a robot, a comparable mean time was reported to be 195 ± 93 seconds^18^. A possible explanation is that the use of a navigated robot reduces time spent on making repeated adjustments to follow the planned path through soft tissue and bone. It also highlights the ease of use of the system, since it only requires placement of the Jamshidi needle into the robot’s instrument guide and then pressing a button to achieve an optimal trajectory without any other adjustments or manual fine tuning. Furthermore, our insertion times are also relatively short compared to other robotic systems. In studies reporting on comparable parts of the surgery, mean time per pedicle cannulation was 202–257 seconds^31,35^. These times do not entirely represent the time per pedicle screw, as no screw was inserted. However, it does indicate that the surgical workflow can be optimized, without much familiarity with the system, to achieve a short surgical time per treated level. The total screw insertion time using ARSN in a clinical study without robot was 5.2 minutes^15^. We believe that using the robot will shorten this time.

Typically, radiation exposure is greater in MIS procedures compared to open spine procedures to both the surgeon, patient, and OR personnel^45,46^. Imaging in combination with computer assisted navigation can decrease radiation exposure to the staff in MIS procedures^45^. In this study, no lead aprons were used as all use of radiation was done with all staff outside of the OR. Meanwhile, the patient radiation exposure from the CBCT with the ARSN system is lower than the radiation dose from comparable intraoperative 3-dimensional image systems^22,47^.

### Limitations

Limitations to our study include the small sample size, lack of a control group and that no actual pedicle screws were placed. However, a K-wire placed through a correctly positioned Jamshidi needle reasonably results in a correct screw placement irrespective of which surgical method is used. Another limitation is that the study was performed on cadavers as opposed to patients but this was considered necessary as a first step to validate the technology. One draw-back with this study design is that there are no movements during surgery due to ventilation of the patient. However, previous studies have shown that the navigation system performs well in live patients due to the use of skin markers for tracking the patient’s movements^15,21^.

When comparing the results of this study to previous studies, using the same navigation system but without robotic guidance, we found statistically superior accuracy in favor of the robotic setup in all comparisons. However, these studies contain dissimilarities which may influence the results and their interpretation. The first study was a cadaveric MIS, using path navigation without instrument tracking^17^, the second, open surgery in a series of deformity cases also using path tracking^15^, and the last, a cadaveric porcine MIS using the added feature of instrument tracking^18^. Meanwhile, the present study used a combination of instrument tracking and robotic guidance in a cadaveric setup. Nonetheless, the comparisons concern technical accuracy, and all studies used the same tracking, planning and imaging system. Therefore, we argue that the comparison, and the statistical analysis, can be justified.

## Conclusions

Using the presented novel robotic guidance system, integrated with an ARSN system in a hybrid OR, for pedicle cannulation, is feasible and provides a very high technical and clinical accuracy.

## Data Availability

The datasets generated and analyzed during the current study are available from the corresponding author on reasonable request.
